# Molecular eidemiology of carbapenem-resistant *Enterobacter cloacae* complex in a tertiary hospital in Shandong, China

**DOI:** 10.1186/s12866-023-02913-x

**Published:** 2023-07-05

**Authors:** Shengnan Hu, Wenyan Xie, Qiwen Cheng, Xiaoning Zhang, Xiutao Dong, Huaiqi Jing, Jiazheng Wang

**Affiliations:** 1 Department of Clinical Laboratory Medicine, The First Affiliated Hospital of Shandong First Medical University & Shandong Provincial Qianfoshan Hospital, Shandong Medicine and Health Key Laboratory of Laboratory Medicine, Jinan, Shandong China; 2 Biodesign Center for Health Through Microbiomes, Arizona State University, Tempe, AZ 85287 USA; 3 State Key Laboratory for Infectious Disease Prevention and Control, National Institute for Communicable Disease Control and Prevention, Changping, Beijing, 102206 People’s Republic of China

**Keywords:** *Enterobacter cloacae* complex, Carbapenemase, NDM-5, NDM-1, ST171, ST418

## Abstract

**Background:**

The increasing incidence and prevalence of carbapenem-resistant *Enterobacter cloacae* complex (CREC) poses great challenges to infection prevention and disease treatment. However, much remains unknown about the clinical characteristics of CREC isolates. Our objective was to characterize antimicrobial resistance and, carbapenemase production in CREC with 36 CREC isolates collected from a tertiary hospital in Shandong, China.

**Results:**

Three types of carbapenemases (NDM, IMP and VIM) were detected in these isolates. Among them, NDM carbapenemases were most prevalent, with a 61.2% (22/36) detection rate for NDM-1, 27.8% (10/36) for NDM-5 and 2.8% (1/36) for NDM-7. IMP-4 was found in two isolates and VIM-1 in only one isolate. The MLST analysis identified 12 different sequence types (STs), of which ST171 (27.8%) was the most prevalent, followed by ST418 (25.0%). ST171 isolates had significantly higher rates of resistance than other STs to gentamicin and tobramycin (*P*s < 0.05), and lower rates of resistance to aztreonam than ST418 and other STs (*Ps* < 0.05). Among 17 carbapenemase-encoding genes, the *bla*_NDM−5_ gene was more frequently detected in ST171 than in ST418 and other isolates (*Ps* < 0.05). In contrast, the *bla*_NDM−1_ gene was more frequently seen in ST418 than in ST171 isolates. One novel ST (ST1965) was identified, which carried the *bla*_NDM−1_ gene.

**Conclusion:**

NDM-5 produced by ST171 and NDM-1 carbapenemase produced by ST418 were the leading cause of CREC in this hospital. This study enhances the understanding of CREC strains and helps improve infection control and treatment in hospitals.

**Supplementary Information:**

The online version contains supplementary material available at 10.1186/s12866-023-02913-x.

## Background

*Enterobacter cloacae* complex comprises several species, including *E. cloacae*, *E. asburiae*, *E. hormaechei*, *E. kobei*, *E. ludwigii* and *E. nimipressuralis* [1, 2]. As Gram-negative opportunistic pathogens, they can cause several diseases such as minor infections of the skin, urinary tract infections, pneumonia and bloodstream infections (BSI) [2]. Due to the unreasonable use of antibiotics, multidrug-resistant *E. cloacae* complex isolates have emerged and spread worldwide [3].

Carbapenems are regarded as the most effective antibiotics against many multidrug-resistant bacteria [4]. However, in recent years, carbapenem-resistant *E. cloacae* complex (CREC) isolates have been increasingly detected from clinical investigations, raising global public health concerns [5]. The production of carbapenemases was the most predominant mechanism associated with carbapenem resistance for the *E. cloacae* complex. Carbapenemases are members of class A, class B and class D β-lactamases [6, 7]. Among them, class A and class D carbapenemases are serine carbapenemases, including KPC, IMI, SME, GES, OXA-23, OXA-24, OXA-48 and OXA-58. Class B carbapenemases are metallo-β-lactamases which mainly include NDM, IMP, VIM, AIM, DIM, GIM and SPM [7]. In addition, some extended-spectrum β-lactamases (ESBLs), such as CTX-M, TEM and SHV, also lead to resistance of *E. cloacae* complex isolates to most β-lactam drugs, posing great challenges for clinical treatment [8].

The multilocus sequence typing (MLST) method has been widely utilized to trace CREC strains worldwide. Previous molecular epidemiological studies have found that the most abundant sequence types (STs) for CREC isolates were ST510 in Cali, Colombia [9], ST74 in Spain [10] and ST171 in the United States [11, 12]. In China, ST418 was previously reported as the predominant ST in Shenzhen [13], ST120 ST in Henan [14], ST190 in Wenzhou [15], ST93 in Liaoning [16] and ST544 in Ningxia [17]. However, until now, scarce data on CREC isolates have been available from Shandong, China.

In this study, we collected and characterized 36 CREC strains over a time span of five years (2018–2022) at a tertiary hospital in Shandong, China. Our work contributes to the understanding of the epidemiology and carbapenem resistance of *E. cloacae* complex strains.

## Methods

### Bacterial isolates

A total of 36 nonrepetitive CREC isolates were obtained from different departments (ICU, urinary surgery ward and other wards) at the First Affiliated Hospital of Shandong First Medical University (Shandong, China). These samples were obtained from 2018 to 2022. All isolates were identified using MALDI-TOF MS (Bruker) and further verified by PCR targeting 16 S rRNA [18]. PCR products were sequenced by Tsingke BioTech Co., Ltd., followed by sequence alignment on the NCBI database.

### Antimicrobial susceptibility test

To test susceptibility, all CREC isolates were exposed to 16 antibiotics, including piperacillin/tazobactam, cefazolin, cefotetan, ceftazidime, ceftriaxone, cefepime, aztreonam, ertapenem, imipenem, amikacin, gentamicin, tobramycin, ciprofloxacin, levofloxacin, nitrofurantoin, and trimethoprim/sulfamethoxazole, by using a Vitek 2 compact system (bioMérieux, Marcy, France) with AST-GN-13 cards. The results were evaluated according to the Clinical and Laboratory Standards Institute (CLSI) criteria.

### mCIM test

To screen for suspected carbapenemase production in the 36 CREC strains, the modified carbapenem inactivation method (mCIM) was performed based on the CLSI guidelines.

### Detection of resistance genes

The whole genomes of the 36 CREC strains were extracted using the DNA nucleic acid extraction kit (Tiangen, China). To detect resistance genes in CREC strains, PCR assays were carried out using conventional PCR amplification [9, 19–21]. The target resistance genes included the carbapenemase gene (*bla*_NDM_, *bla*_VIM_, *bla*_IMP_, *bla*_KPC_, *bla*_SPM_, *bla*_IMI_, *bla*_OXA−23_, *bla*_OXA−24_, *bla*_OXA−48_, *bla*_OXA−58_, *bla*_SIM_, *bla*_DIM_, *bla*_BIC_, *bla*_GIM_, *bla*_SME_, *bla*_AIM_, and *bla*_GES_) and the extended-spectrum β-lactamase genes (*bla*_CTX−M_, *bla*_SHV_, and *bla*_TEM_). Positive amplicons were sequenced by Tsingke BioTech Co., Ltd. in both directions. The sequences were analyzed against the NCBI database by the Basic Local Alignment Search Tool (BLAST).

### Multilocus sequence typing (MLST)

MLST analyses were performed for all CREC isolates as described previously (https://pubmlst.org/organisms/enterobacter-cloacae/). The amplified fragments of seven housekeeping genes (*dnaA*, *fusA*, *gyrB*, *leuS*, *pyrG*, *rplB*, and *rpoB*) were sequenced in both directions. The sequences were aligned with the reference sequence from the MLST database. Newly identified STs were submitted to the MLST database curator for approval, and new numbers were assigned. A minimum-spanning tree using the allelic difference between isolates of the seven housekeeping genes was constructed using BioNumerics software.

### Statistical analysis

Statistical analyses were performed using SPSS Statistics 21.0 for Windows. A two-sided p value of less than 0.05 was considered statistically significant.

## Results

### Clinical and demographic characteristics of CREC isolates

The clinical characteristics of the 36 CREC isolates are shown in Table 1. A total of 36 nonduplicate CREC isolates were collected from 2018 to 2022. Among them, one was collected in 2018, seven in 2019, three in 2020, 18 in 2021 and seven in 2022. The isolates were primarily from urine (n = 10, 27.8%), sputum (n = 7, 19.4%) and blood (n = 6, 16.7%) specimens. No more than three isolates were found in each of other specimens. The isolates were primarily collected from the ICU (n = 16, 44.4%), followed by the urinary surgery ward (n = 4, 11.1%). No more than three isolates were from each of the other hospital wards.

**Table 1:** Microbiological and molecular characteristics of 36 CREC isolates

Isolate	Year of isolation	Gender/Age (Years)	Ward	Specimen	ST	Carbapenemase	ESBL
CREC01	2018	M/48	Hepatobiliary surgery	dr	1120	NDM-1	TEM
CREC02	2019	M/50	ICU	sp	171	NDM-5	TEM
CREC03	2019	M/51	Neurosurgery	ca	171	NDM-5	TEM
CREC04	2019	M/87	ICU	bl	418	NDM-1	TEM
CREC05	2019	F/27	Urinary surgery	ps	336	NDM-1	TEM, CTX-M
CREC06	2019	M/62	ICU	sp	418	NDM-1	TEM
CREC07	2019	F/81	ICU	sp	25	NDM-1	TEM, CTX-M
CREC08	2019	M/86	Respiratory medicine	sp	418	NDM-1	TEM
CREC09	2020	F/31	orthopedic	se	97	IMP-4	
CREC10	2020	M/36	Neurosurgery	sp	1965	NDM-1	TEM
CREC11	2020	F/67	Hepatobiliary surgery	dr	97	IMP-4	
CREC12	2021	F/81	ICU	bl	564	VIM-1	
CREC13	2021	M/65	Urinary surgery	ur	231	NDM-1	TEM, CTX-M
CREC14	2021	F/82	ICU	bl	171	NDM-5	TEM
CREC15	2021	F/61	Cardiovasology	bl	113	NDM-1	TEM, CTX-M
CREC16	2021	M/64	Cardiovasology	bl	113	NDM-1	TEM, CTX-M
CREC17	2021	M/58	ICU	sp	418	NDM-1	TEM
CREC18	2021	M/50	Neurosurgery	bl	171	NDM-5	TEM
CREC19	2021	M/76	Hepatobiliary surgery	pf	171	NDM-7	TEM
CREC20	2021	M/77	Urinary surgery	ur	231	NDM-1	
CREC21	2021	M/56	Gastroenterology	ur	127	NDM-1	
CREC22	2021	M/54	Gastroenterology	ca	171	NDM-5	TEM
CREC23	2021	M/60	ICU	ur	171	NDM-5	TEM
CREC24	2021	M/57	orthopedic	se	171	NDM-1	TEM, CTX-M
CREC25	2021	M/79	ICU	pf	418	NDM-1	TEM
CREC26	2021	M/77	Urinary surgery	ur	231	NDM-1	TEM, CTX-M
CREC27	2021	M/77	Nephrology	ur	127	NDM-1	TEM
CREC28	2021	M/61	ICU	se	418	NDM-1	TEM
CREC29	2021	M/56	ICU	ts	418	NDM-1	TEM
CREC30	2022	M/4	Hematology	sp	316	NDM-1	
CREC31	2022	F/83	ICU	ts	231	NDM-5	
CREC32	2022	F/38	General Practice	ur	171	NDM-5	TEM
CREC33	2022	M/81	ICU	ca	418	NDM-1	TEM
CREC34	2022	M/61	ICU	ur	418	NDM-1	
CREC35	2022	M/79	ICU	ur	231	NDM-5	
CREC36	2022	F/59	ICU	ur	171	NDM-5	TEM

CREC: carbapenem-resistant *Enterobacter cloacae* complex, M: Male; F: Female; ICU: Intensive Care Unit; bl: blood; ca.: catheter; dr: drainage; pf: peritoneal fluid; ps: preservation solution; se: secretion; sp: sputum; ts: throat swab; ur: urine

The demographic characteristics of the 36 CREC isolates are shown in Table 1 and summarized in Table 2. Briefly, 72.2% (26 of 36) of the infected patients were male and the rest were female. 58.3% (21 of 36) of them were older adults aged 60 years and over, 27.8% (10 of 36) middle-aged adults aged 41–60 and 2.8% (1 of 36) teenagers younger aged 12–20.

**Table 2:** Demographic characteristics corresponding of 36 CREC isolates

	Characteristic	n (%)
Age	Teenagers (12–20 years)	1 (2.8%)
	Young adult (21–40 years)	4 (11.1%)
	Middle adult (41–60 years)	10 (27.8%)
	Elderly (> 60 years)	21 (58.3%)
Gender	Male	26(72.2%)
	Female	10 (27.8%)

### Antibiotic susceptibility

The antimicrobial susceptibility profiles of 36 CREC strains are shown in Table 3 and Additional file 1. In general, all CREC isolates were resistant to cefzolin, ceftetam, ceftazidime, ceftriaxone ertapenem and imipenem. Most isolates (over 86.1%) were also resistant to piperacillin/tazobactam, cefepime, ciprofloxacin and trimethoprim/sulfamethoxazole. In addition, 72.2% of the isolates were resistant to levofloxacin, 52.8% isolates resistant to aztreonam and gentamicin, and 41.7% to macrodantin. In contrast, the resistance rates for tobramycin and amikacin were only 22.2% and 2.8%, respectively.

**Table 3:** Resistance rates for ST171 and ST418 isolates

Antimicrobial	Resistance rate (%)					Resistance rate (%)		
	Overall	ST171	Other STs	P-value		ST171	ST418	P-value
	n = 36	n = 10	n = 26			n = 10	n = 9	
Piperacillin/tazobactam	94.4%	100.0%	92.3%	1.000		100.0%	100.0%	-
Cefazolin	100.0%	100.0%	100.0%	-		100.0%	100.0%	-
Cefotetan	100.0%	100.0%	100.0%	-		100.0%	100.0%	-
Ceftazidime	100.0%	100.0%	100.0%	-		100.0%	100.0%	-
Ceftriaxone	100.0%	100.0%	100.0%	-		100.0%	100.0%	-
Ertapenem	100.0%	100.0%	100.0%	-		100.0%	100.0%	-
Imipenem	100.0%	100.0%	100.0%	-		100.0%	100.0%	-
Cefepime	88.9%	100.0%	84.6%	0.559		100.0%	100.0%	-
Ciprofloxacin	88.9%	100.0%	84.6%	0.559		100.0%	100.0%	-
Trimethoprim/ sulfamethoxazole	86.1%	100.0%	80.8%	0.293		100.0%	88.9%	0.474
Levofloxacin	72.2%	80.0%	69.2%	0.689		80.0%	100.0%	0.474
Aztreonam	52.8%	20.0%	65.4%	0.025		20.0%	100.0%	0.001
Gentamicin	52.8%	90.0%	38.5%	0.008		90.0%	77.8%	0.582
Nitrofurantoin	41.7%	30.0%	46.2%	0.468		30.0%	55.6%	0.370
Tobramycin	22.2%	60.0%	7.7%	0.002		60.0%	22.2%	0.170
Amikacin	2.8%	10.0%	0.0%	0.278		10.0%	0.0%	1.000

### Distribution of carbapenemase- and ESBL-encoding genes

All 36 CREC strains showed positive phenotypes as detected by the modified carbapenem inactivation method (mCIM) indicating that they were carbapenemase producers. All of them harbored carbapenemase-encoding genes. Three types of carbapenemases (NDM, IMP and VIM) were detected in these isolates. Among them, the *bla*_NDM_ gene was the most prevalent carbapenemase**-**encoding gene, with a 61.2% (22/36) detection rate for the *bla*_NDM−1_ gene, 27.8% (10/36) for the *bla*_NDM−5_ gene and 2.8% (1/36) for the *bla*_NDM−7_ gene. The *bla*_IMP_ gene was only found in only two isolates (5.6%), and both were *bla*_IMP−4_. In addition, we found the *bla*_VIM−1_ gene in only one isolate (2.8%). None of these isolates had two or more carbapenemase**-**encoding genes, and none of them had other carbapenemase**-**encoding genes tested in this study.

Twenty of the 36 CREC isolates harbored the extended-spectrum β-lactamase (ESBL) genes. Among them, the *bla*_TEM_ gene was the most prevalent, with a 52.8% (19/36) detection rate, followed by the *bla*_CTX−M_ gene in seven isolates (19.4%). None of them harbored *bla*_SHV_ gene.

### MLST profile

**Fig. 1:**
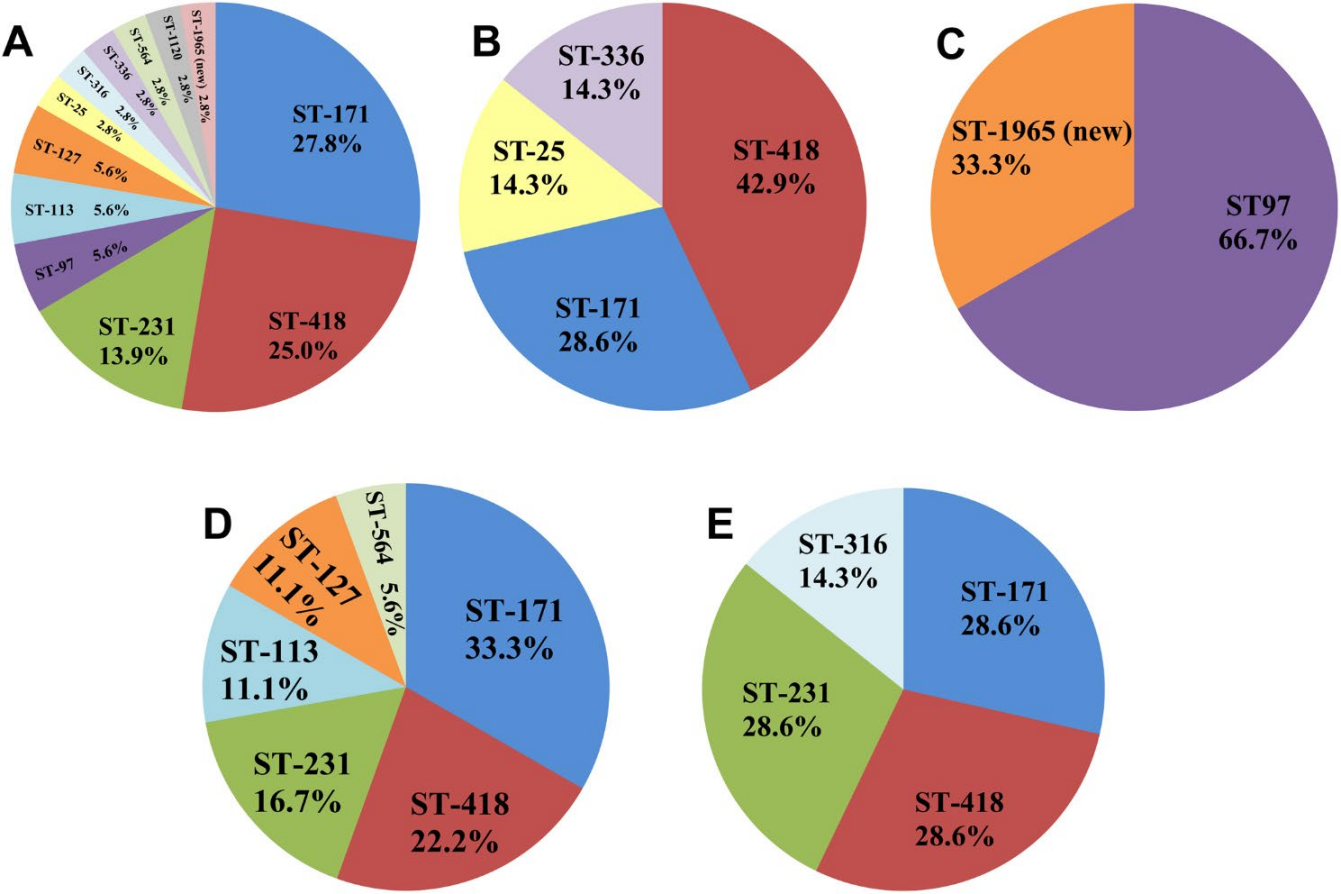
MLST population analysis over the different years. **(A)** STs in all 36 isolates. **(B)** STs distribution in 2019. **(C)** STs distribution in 2020. **(D)** STs distribution in 2021. **(E)** STs distribution in 2022

The MLST analysis revealed a total of 12 different STs, including 11 existing STs and one novel ST identified in this study. The new ST was submitted for ST assignment, which was ST1965. The profiles of the newly identified STs are listed in Table 4. In the alignment of the MLST sequence, a novel sequence was found in *pyrG*, which was designated as *pyrG*-461. Moreover, 83.3% (30/36) of the isolates were represented by six main STs (having ≥ 2 isolates. The most prevalent ST was ST171, which accounted for 27.8% (10/36) of the isolates, followed by ST418 accounting for 25.0% (9/36) (Fig. 1 and Additional file 2).

**Table 4:** Allelic profiles of the new ST found in this study

ST	*dnaA*	*fusA*	*gyrB*	*leuS*	*pyrG*	*rplB*	*rpoB*
ST-1965	46	20	20	96	461	29	54

ST171 and ST418 were the most predominant STs found in 2019, 2021 and 2022; however, in 2020 these two STs were not detected and the dominant ST was ST97. In addition, both ST97 isolates harbored the *bla*_IMP−4_ gene, with the detection rate higher than that of the other STs (P < 0.05) (Table 5). For other minor STs, the distributions varied across years, with some STs diminishing or switching to another minor ST (Fig. 1). For instance, ST231 isolates were not observed in 2019 and 2020. However, in 2021, the proportion of this ST increased to 22.2% in 2021 and 28.6% in 2022.

**Table 5:** Prevalence of carbapenem resistance genes among ST171 and ST418 isolates

gene	distributing in				distributing in		
	ST171(n = 10)	ST418(n = 9)	P-value		ST171(n = 10)	Other STs(n = 26)	P-value
	n (%)	n (%)			n (%)	n (%)	
*bla*_NDM−1_	1(10.0%)	9(100.0%)	0.000		1(10.0%)	21(80.8%)	0.000
*bla*_NDM−5_	8(80.0%)	0(0.0%)	0.001		8(80.0%)	2(7.7%)	0.000
*bla*_NDM−7_	1(10.0%)	0(0.0%)	1.000		1(10.0%)	0(0.0%)	0.278
*bla*_IMP−4_	0(0.0%)	0(0.0%)	-		0(0.0%)	2(7.7%)	1.000
*bla*_VIM−1_	0(0.0%)	0(0.0%)	-		0(0.0%)	1(3.8%)	1.000

One clonal complex (CC) and 10 singletons were identified, which suggested high genetic diversity. CC231 (accounting for six isolates) was the only CC that contained ST231 and the new ST1965 (Table 4). In addition, the other 10 individual STs were all singletons which accounted for 30 isolates. The detailed MLST profiles are presented in Additional file 2.

### Comparisons of resistance and carbapenemase-encoding genes between ST171 and ST418

As described in Tables 3 and 90% and 60% of ST171 isolates were resistant to gentamicin and tobramycin, respectively, which was significantly higher than other STs isolates (P < 0.05). In contrast, only 20% of ST171 isolates were resistant to aztreonam which was significantly lower than ST418 and other STs isolates (both P < 0.05). No significant differences were found in the resistance to other antibiotics between ST171 and ST418 isolates or between ST171 and other STs. Among the 17 carbapenemase-encoding genes tested in this study, 80% ST171 (8 of 10) isolates harbored *bla*_NDM−5_ gene which was more frequent than ST418 isolates (0%, P < 0.05) as well as the other STs (7.7%, P < 0.05). In contrast, all ST418 isolates and 80.8% of the other ST isolates were positive for the *bla*_NDM−1_ gene. The detection rates were significantly higher than those of the ST171 isolates (0%, P < 0.05). No significant differences were found in the positive rates for the remaining carbapenemase-encoding genes between these two types of strains (Table 5).

## Discussion

CREC isolates have been discovered in many countries and thus become a global health threat [13, 22–24]. Herein, we characterized the epidemiology and carbapenem resistance mechanisms of 36 CREC strains in a tertiary hospital in Shandong, China from 2018 to 2022.

Resistance to carbapenems is associated with several mechanisms. Among them, carbapenemase production is the main drug resistance mechanism. Carbapenemases belong to three classes of β-lactamases: Ambler class A, B, and D β-lactamases [25]. Class B β-lactamases are metallo β-lactamases (MBLs). As they can catalyze the hydrolysis of nearly all available β-lactam antibiotics, MBL-type carbapenemases present obstacles for clinical treatments [26]. New Delhi metallo-β-lactamase (NDM), IMP and VIM are three important acquired MBLs [27, 28]. Among them, NDM is encoded by the *bla*_NDM_ gene and was first detected in *Klebsiella pneumoniae* in 2008 in India [29]. Although the production of *Klebsiella pneumoniae* carbapenemase (KPC) producing *Enterobacteriaceae* is widespread globally [30] and this mechanism also accounts for the majority of CREC isolates in the United States [11, 12] and Colombia [9], none of the CREC isolates tested in our study harbored the KPC gene. In contrast, 91.7% (33/36) of these CREC isolates carried the *bla*_NDM_ gene in the present study, which suggested that the *bla*_NDM_ gene was the predominant mechanism of carbapenem resistance. The detection rate was higher than that in the investigation by Jin’s group, who detected the resistance determinants of 55 CREC strains isolated from 11 Chinese cities and found that 36 of them were *bla*_NDM_ positive [13]. This gene was also frequently detected in other cities in China. For example, the prevalence of *bla*_NDM_ was 72.7% (8 of 11) in Henan [14], 50% in Shenyang (9 of 18) [16], 16.7% in Ningxia (2 of 12) [17] and 17.7% in Wenzhou (20 of 113) [15]. Besides, in this study, all NDM-producing strains were resistant to piperacillin/tazobactam, cefzolin, ceftetam, ceftazidime, ceftriaxone ertapenem and imipenem. The non-sensitive rates to cefepime, ciprofloxacin and trimethoprim/sulfamethoxazole, levofloxacin, gentamicin and macrodantin were also higher than 69.7%. In contrast, 97.0% of NDM-producing strains showed amikacin sensitive phenotype indicating that amikacin would be a therapeutic agent to control NDM-producing *E. cloacae* complex infections.

IMP-type carbapenemases have been reported globally [6, 31] and have become the most predominant form in Australia [24, 32, 33]. IMP-4 carbapenemases are the most predominant IMP subtypes in the world [33, 34]. In the present study, IMP-type carbapenemase was found to be the second most common carbapenemase (5.6%, 2 of 36). Besides, both IMP-type carbapenemases in this study were identified as IMP-4, which was consistent with the worldwide distribution. VIM-producing CREC are mainly detected in Spain and some other European countries [35]. However, in the present study, we only identified one VIM-1 carbapenemase-producing strain. This result was similar to some other studies in China, which also showed that the detection rate of the VIM gene in *Enterobacteriaceae* is very low in China [36].

A total of 12 STs were found in this study. Among them, ST171 was the dominant ST accounting for 27.8% of the strains, followed by ST418 accounting for 25.0%. Although ST171 was rare in global surveys [3, 5, 37, 38], it has been identified as a major ST among all CREC isolates with epidemic potential in the United States [5, 12, 39]. Previous studies also indicated that ST171 CREC isolates were primarily associated with *bla*_KPC−3_, followed by *bla*_KPC−2_ and *bla*_KPC−4_ [11]. In the present study, we also observed that ST171 was the most abundant ST among all CREC isolates, which was surprisingly different from other regions of China but consistent with the United States. Notably, unlike the major epidemic strain ST171 in the United States, which primarily produces KPC carbapenemases, all the ST171 isolates in this study were NDM-producing strains. Considering the local transmission and clonal expansion of ST171 in the United States, close attention should be paid to prevent the spread of high-risk clones. Interestingly, in this study, we also found that 80.0% of the ST171 (8 of 10) isolates harbored the *bla*_NDM−5_ gene, whereas most (80.8%) of the remaining isolates were *bla*_NDM−1_ positive bacteria and only two (7.7%) of them contained the *bla*_NDM−5_ gene which suggested that ST171 isolates may tend to acquire NDM-5 carbapenem resistance determinants. However, further investigation is required to explore the reason of the high correlation between the ST171 sequence type and *bla*_NDM−5_ gene.

ST418 isolates have emerged in several cities of China, such as Nanjing, Shanghai, Shenzhen and Guangdong [13, 40–42]. In Shenzhen and Guangdong, this ST served as the most common genotype [13, 42]. Besides, previous studies also found that ST418 was the main epidemic type of NDM-1-producing CREC isolates in these cities of China [13]. In the present study, ST418 was found in 25% of the CREC isolates and was the second most abundant ST among all CREC isolates. Moreover, unlike ST171 isolates, which tend to harbor the *bla*_NDM−5_ gene, all isolates were positive for the *bla*_NDM−1_ gene. These results were consistent with previous studies of these cities in China [13].

We also observed one new ST (ST1965) in this study, and it was classified into CC231, which suggested that *E. cloacae* complex isolates were diverse and still in clonal expansion. Besides, we found that this new ST isolate harbored the *bla*_NDM−1_ gene. To our knowledge, this is the first report in the world of ST1965 carbapenem-resistant *E. cloacae* complex isolate carrying the *bla*_NDM−1_ gene. Although this new ST was in the minority, the isolate within it may give rise to future disease outbreaks; therefore, close attention should be paid to this new ST to identify and further limit both transmission and outbreaks.

## Conclusions

In our study, we characterized the molecular epidemiology and carbapenem-resistance mechanisms of *E. cloacae* complex strains in a tertiary hospital in Shandong, China. NDM-5 carbapenemase produced by ST171 and NDM-1 carbapenemase produced by ST418 were the leading cause for the carbapenem resistance of *E. cloacae* complex strains in this hospital. One novel ST (ST1965) was detected, and this new ST isolate carried the *bla*_NDM−1_ gene. This study contributes to a better understanding of CREC strains and improves infection control and treatment in hospitals.

## Electronic supplementary material

Below is the link to the electronic supplementary material.

Supplementary Material 1

Supplementary Material 2

## Data Availability

The datasets used and/or analyzed during the current study are within the manuscript and the Additional files. The sequences analysed during the current study were deposited in the GenBank database (accession numbers: OP806578-OP806829 and OP806908-OP806970).

## Abbreviations

CREC: carbapenem-resistant *Enterobacter cloacae* complex
PCR: polymerase chain reaction
MLST: Multilocus sequence typing
ST: sequence type
BSI: Bloodstream infection
ESBLs: extended-spectrum β-lactamases
MBLs: metallo β-lactamases
NDM: New Delhi metallo-β-lactamase
KPC: *Klebsiella pneumoniae* carbapenemase
BLAST: Basic Local Alignment Search Tool
CC: Clonal complex
CLSI: Clinical and Laboratory Standards Institute
MIC: minimum inhibitory concentration
MALDI-TOF MS: matrix assisted laser desorption ionization time of flight mass spectrometry
