# Supplementary material for: Molecular eidemiology of carbapenem-resistant Enterobacter cloacae complex in a tertiary hospital in Shandong, China
Source: BMC Microbiol. 2023 Jul 5;23:177. doi: 10.1186/s12866-023-02913-x (PMC10320948; doi:10.1186/s12866-023-02913-x)
Supplement: Supplementary file 1 — Supplementary Material 1 [file 12866_2023_2913_MOESM1_ESM.docx]

Table S1 Minimum Inhibitory Concentrations (MICs) of the tested antimicrobial agents

TZP: piperacillin/tazobactam; CZO: Cefazolin; CTT: Cefotetan; CAZ: ceftazidime; CRO: ceftriaxone; FEP: cefepime; ATM: aztreonam; ETP: ertapenem; IPM: imipenem; AMK, amikacin; GEN: gentamicin; TOB: tobramycin; CIP: ciprofloxacin; LEV: levofloxacin; NIT: nitrofurantoin; SXT: sulfamethoxazole/trimethoprim.

| **strain** | **TZP** | **CZO** | **CTT** | **CAZ** | **CRO** | **FEP** | **ATM** | **ETP** | **IPM** | **AMK** | **GEN** | **TOB** | **CIP** | **LEV** | **NIT** | **SXT** |
| --- | --- | --- | --- | --- | --- | --- | --- | --- | --- | --- | --- | --- | --- | --- | --- | --- |
| CREC-01 | ≥128 | ≥64 | ≥64 | ≥64 | ≥64 | ≥64 | ≥64 | ≥8 | ≥16 | ≤2 | ≥16 | 4 | ≥4 | ≥8 | 64 | ≥320 |
| CREC-02 | ≥128 | ≥64 | ≥64 | ≥64 | ≥64 | ≥64 | ≤1 | ≥8 | ≥16 | ≤2 | ≥16 | 8 | ≥4 | ≥8 | 128 | ≥320 |
| CREC-03 | ≥128 | ≥64 | ≥64 | ≥64 | ≥64 | 32 | ≤1 | ≥8 | ≥16 | ≤2 | ≥16 | ≥16 | ≥4 | 1 | 64 | ≥320 |
| CREC-04 | ≥128 | ≥64 | ≥64 | ≥64 | ≥64 | ≥64 | ≥64 | ≥8 | 8 | ≤2 | ≥16 | 8 | ≥4 | ≥8 | 64 | ≥320 |
| CREC-05 | 64 | ≥64 | ≥64 | ≥64 | ≥64 | ≥64 | 4 | 4 | 4 | ≤2 | 8 | 8 | 1 | 1 | 32 | ≥320 |
| CREC-06 | ≥128 | ≥64 | ≥64 | ≥64 | ≥64 | ≥64 | ≥64 | ≥8 | ≥16 | ≤2 | ≥16 | 8 | ≥4 | ≥8 | 128 | ≥320 |
| CREC-07 | ≥128 | ≥64 | ≥64 | ≥64 | ≥64 | ≥64 | ≥64 | ≥8 | ≥16 | ≤2 | 4 | 4 | ≤0.25 | ≤0.25 | 32 | ≤20 |
| CREC-08 | ≥128 | ≥64 | ≥64 | ≥64 | ≥64 | ≥64 | ≥64 | ≥8 | ≥16 | ≤2 | ≤1 | ≤1 | ≥4 | ≥8 | 128 | ≥320 |
| CREC-09 | 8 | ≥64 | ≥64 | ≥64 | ≥64 | 8 | ≤1 | ≥8 | 4 | ≤2 | ≤1 | ≤1 | 1 | 1 | 64 | ≤20 |
| CREC-10 | ≥128 | ≥64 | ≥64 | ≥64 | ≥64 | ≥64 | ≥64 | ≥8 | ≥16 | ≤2 | ≥16 | 8 | ≥4 | ≥8 | 128 | ≥320 |
| CREC-11 | 8 | ≥64 | ≥64 | ≥64 | 32 | 8 | ≤1 | ≥8 | ≥16 | ≤2 | ≤1 | ≤1 | 1 | 1 | 64 | ≤20 |
| CREC-12 | 64 | ≥64 | ≥64 | ≥64 | ≥64 | 32 | ≥64 | 4 | 4 | ≤2 | 4 | 8 | ≥4 | ≥8 | 64 | ≥320 |
| CREC-13 | ≥128 | ≥64 | ≥64 | ≥64 | ≥64 | ≥64 | ≥64 | ≥8 | ≥16 | ≤2 | ≤1 | ≤1 | ≥4 | ≥8 | ≥512 | ≤20 |
| CREC-14 | ≥128 | ≥64 | ≥64 | ≥64 | ≥64 | ≥64 | ≤1 | ≥8 | ≥16 | ≤2 | ≥16 | ≥16 | ≥4 | ≥8 | 128 | ≥320 |
| CREC-15 | 64 | ≥64 | ≥64 | ≥64 | ≥64 | ≥64 | 4 | ≥8 | 8 | ≤2 | 8 | 8 | 0.5 | 1 | 64 | ≥320 |
| CREC-16 | 64 | ≥64 | ≥64 | ≥64 | ≥64 | ≥64 | 4 | ≥8 | 8 | ≤2 | 8 | 8 | 0.5 | 1 | 64 | ≥320 |
| CREC-17 | ≥128 | ≥64 | ≥64 | ≥64 | ≥64 | ≥64 | ≥64 | ≥8 | ≥16 | ≤2 | ≥16 | 8 | ≥4 | ≥8 | 64 | ≥320 |
| CREC-18 | ≥128 | ≥64 | ≥64 | ≥64 | ≥64 | 32 | ≤1 | ≥8 | ≥16 | ≤2 | ≥16 | ≥16 | ≥4 | ≥8 | 64 | ≥320 |
| CREC-19 | ≥128 | ≥64 | ≥64 | ≥64 | ≥64 | ≥64 | 16 | ≥8 | ≥16 | ≤2 | ≤1 | 8 | ≥4 | ≥8 | 128 | ≥320 |
| CREC-20 | ≥128 | ≥64 | ≥64 | ≥64 | ≥64 | ≥64 | ≥64 | ≥8 | ≥16 | ≤2 | ≤1 | 8 | ≥4 | ≥8 | 128 | ≥320 |
| CREC-21 | 64 | ≥64 | ≥64 | ≥64 | ≥64 | 4 | ≤1 | 4 | 8 | ≤2 | ≤1 | ≤1 | 0.5 | 1 | 64 | ≥320 |
| CREC-22 | ≥128 | ≥64 | ≥64 | ≥64 | ≥64 | ≥64 | ≤1 | ≥8 | ≥16 | ≤2 | ≥16 | ≥16 | ≥4 | ≥8 | 64 | ≥320 |
| CREC-23 | ≥128 | ≥64 | ≥64 | ≥64 | ≥64 | 32 | ≤1 | ≥8 | ≥16 | ≤2 | ≥16 | ≥16 | ≥4 | ≥8 | 64 | ≥320 |
| CREC-24 | 64 | ≥64 | ≥64 | ≥64 | ≥64 | 32 | 32 | 4 | 8 | ≥64 | ≥16 | ≥16 | ≥4 | ≥8 | 64 | ≥320 |
| CREC-25 | ≥128 | ≥64 | ≥64 | ≥64 | ≥64 | ≥64 | ≥64 | ≥8 | ≥16 | ≤2 | ≤1 | ≤1 | ≥4 | ≥8 | 64 | ≥320 |
| CREC-26 | ≥128 | ≥64 | ≥64 | ≥64 | ≥64 | ≥64 | ≥64 | ≥8 | ≥16 | ≤2 | ≥16 | 8 | ≥4 | ≥8 | ≥512 | ≥320 |
| CREC-27 | 64 | ≥64 | ≥64 | ≥64 | ≥64 | ≥64 | ≥64 | ≥8 | ≥16 | ≤2 | ≤1 | ≤1 | ≥4 | ≥8 | 256 | ≥320 |
| CREC-28 | ≥128 | ≥64 | ≥64 | ≥64 | ≥64 | ≥64 | ≥64 | ≥8 | ≥16 | ≤2 | ≥16 | 8 | ≥4 | ≥8 | 128 | ≥320 |
| CREC-29 | ≥128 | ≥64 | ≥64 | ≥64 | ≥64 | ≥64 | ≥64 | ≥8 | ≥16 | ≤2 | ≥16 | ≥16 | ≥4 | ≥8 | 64 | ≥320 |
| CREC-30 | 64 | ≥64 | ≥64 | ≥64 | ≥64 | 8 | ≤1 | 4 | 4 | ≤2 | 8 | 8 | 1 | 1 | 64 | ≥320 |
| CREC-31 | ≥128 | ≥64 | ≥64 | ≥64 | ≥64 | ≥64 | ≤1 | ≥8 | ≥16 | ≤2 | ≤1 | 8 | ≥4 | ≥8 | 128 | ≥320 |
| CREC-32 | ≥128 | ≥64 | ≥64 | ≥64 | ≥64 | 16 | ≤1 | ≥8 | ≥16 | ≤2 | ≥16 | 8 | 2 | 1 | 64 | ≥320 |
| CREC-33 | ≥128 | ≥64 | ≥64 | ≥64 | ≥64 | ≥64 | ≥64 | ≥8 | ≥16 | ≤2 | ≥16 | 8 | ≥4 | ≥8 | 128 | ≥320 |
| CREC-34 | ≥128 | ≥64 | ≥64 | ≥64 | ≥64 | ≥64 | ≥64 | ≥8 | ≥16 | ≤2 | ≥16 | ≥16 | ≥4 | ≥8 | 128 | ≤20 |
| CREC-35 | ≥128 | ≥64 | ≥64 | ≥64 | ≥64 | ≥64 | ≤1 | ≥8 | ≥16 | ≤2 | ≤1 | 8 | ≥4 | ≥8 | 128 | ≥320 |
| CREC-36 | ≥128 | ≥64 | ≥64 | ≥64 | ≥64 | ≥64 | ≤1 | ≥8 | ≥16 | ≤2 | ≥16 | 8 | ≥4 | ≥8 | 64 | ≥320 |
