# Supplementary material for: Molecular eidemiology of carbapenem-resistant Enterobacter cloacae complex in a tertiary hospital in Shandong, China
Source: BMC Microbiol. 2023 Jul 5;23:177. doi: 10.1186/s12866-023-02913-x (PMC10320948; doi:10.1186/s12866-023-02913-x)
Supplement: Supplementary file 2 — Supplementary Material 2 [file 12866_2023_2913_MOESM2_ESM.docx]

Table S2 Allelic profiles of the carbapenem-resistant *E. cloacae* complex isolates used in this study

| **Isolate** | ***dnaA*** | ***fusA*** | ***gyrB*** | ***leuS*** | ***pyrG*** | ***rplB*** | ***rpoB*** | **ST** |
| --- | --- | --- | --- | --- | --- | --- | --- | --- |
| CREC-01 | 9 | 4 | 344 | 61 | 89 | 4 | 9 | 1120 |
| CREC-02 | 49 | 21 | 19 | 44 | 45 | 12 | 32 | 171 |
| CREC-03 | 49 | 21 | 19 | 44 | 45 | 12 | 32 | 171 |
| CREC-04 | 53 | 35 | 154 | 44 | 45 | 4 | 6 | 418 |
| CREC-05 | 59 | 9 | 113 | 115 | 70 | 6 | 6 | 336 |
| CREC-06 | 53 | 35 | 154 | 44 | 45 | 4 | 6 | 418 |
| CREC-07 | 24 | 14 | 43 | 52 | 27 | 18 | 21 | 25 |
| CREC-08 | 53 | 35 | 154 | 44 | 45 | 4 | 6 | 418 |
| CREC-09 | 59 | 9 | 62 | 9 | 62 | 25 | 6 | 97 |
| CREC-10 | 46 | 20 | 20 | 96 | 461 | 29 | 54 | 1965 (new ST) |
| CREC-11 | 59 | 9 | 62 | 9 | 62 | 25 | 6 | 97 |
| CREC-12 | 49 | 69 | 20 | 44 | 64 | 4 | 6 | 564 |
| CREC-13 | 46 | 20 | 20 | 96 | 45 | 29 | 54 | 231 |
| CREC-14 | 49 | 21 | 19 | 44 | 45 | 12 | 32 | 171 |
| CREC-15 | 4 | 22 | 68 | 69 | 37 | 4 | 24 | 113 |
| CREC-16 | 4 | 22 | 68 | 69 | 37 | 4 | 24 | 113 |
| CREC-17 | 53 | 35 | 154 | 44 | 45 | 4 | 6 | 418 |
| CREC-18 | 49 | 21 | 19 | 44 | 45 | 12 | 32 | 171 |
| CREC-19 | 49 | 21 | 19 | 44 | 45 | 12 | 32 | 171 |
| CREC-20 | 46 | 20 | 20 | 96 | 45 | 29 | 54 | 231 |
| CREC-21 | 46 | 20 | 74 | 44 | 45 | 24 | 6 | 127 |
| CREC-22 | 49 | 21 | 19 | 44 | 45 | 12 | 32 | 171 |
| CREC-23 | 49 | 21 | 19 | 44 | 45 | 12 | 32 | 171 |
| CREC-24 | 49 | 21 | 19 | 44 | 45 | 12 | 32 | 171 |
| CREC-25 | 53 | 35 | 154 | 44 | 45 | 4 | 6 | 418 |
| CREC-26 | 46 | 20 | 20 | 96 | 45 | 29 | 54 | 231 |
| CREC-27 | 46 | 20 | 74 | 44 | 45 | 24 | 6 | 127 |
| CREC-28 | 53 | 35 | 154 | 44 | 45 | 4 | 6 | 418 |
| CREC-29 | 53 | 35 | 154 | 44 | 45 | 4 | 6 | 418 |
| CREC-30 | 59 | 88 | 82 | 9 | 67 | 6 | 6 | 316 |
| CREC-31 | 46 | 20 | 20 | 96 | 45 | 29 | 54 | 231 |
| CREC-32 | 49 | 21 | 19 | 44 | 45 | 12 | 32 | 171 |
| CREC-33 | 53 | 35 | 154 | 44 | 45 | 4 | 6 | 418 |
| CREC-34 | 53 | 35 | 154 | 44 | 45 | 4 | 6 | 418 |
| CREC-35 | 46 | 20 | 20 | 96 | 45 | 29 | 54 | 231 |
| CREC-36 | 49 | 21 | 19 | 44 | 45 | 12 | 32 | 171 |
